# Symptom-level interactions between digital addiction and psychological resources: a large-scale cross-validation study

**DOI:** 10.3389/fpsyg.2026.1760981

**Published:** 2026-04-16

**Authors:** Abdullah Aldemir

**Affiliations:** Department of Guidance and Psychological Counseling, Tokat Gaziosmanpaşa University, Tokat, Türkiye

**Keywords:** adolescents, digital addiction, network analysis, nomophobia, self-regulation, validation study

## Abstract

**Background:**

Digital addiction in adolescents is often conceptualized as a latent construct, masking the complex interplay between specific symptoms and psychological resources. This study aims to map the specific symptom-level interactions between digital addiction (Social Media Addiction, Nomophobia) and cognitive resources (Self-Regulation, Self-Efficacy) using a large-scale network analysis approach.

**Method:**

The study employed a split-half cross-validation strategy with a total sample of 1,497 adolescents (*M*_age_ = 16.07). The dataset was randomly partitioned into Discovery (*n* = 748) and Validation (*n* = 749) groups. Network structure was estimated using the Gaussian Graphical Model (GGM) with polychoric correlations. Robustness was assessed via Network Comparison Test (NCT), sensitivity analysis controlling for demographics, and case-dropping bootstrapping.

**Results:**

The network analysis revealed a distinct topological separation between addiction symptoms and cognitive resources. Centrality analysis identified “intolerance of environmental restriction” (Nomophobia item d6) as the most influential node in the network (highest EI = 1.81 relative to other nodes). Bridge centrality analysis highlighted “withdrawal upon prohibition” (Social Media Addiction item b5) as the critical bridge as the strongest bridge node (highest BS = 0.61) associating addiction symptoms with cognitive resources. Conversely, “self-monitoring” (a8) emerged as the most prominent inversely associated (BS = 0.47). The network structure was highly consistent across subsamples (NCT: *p* > 0.05), robust to demographic covariates (*r* = 0.98), and stable [CS_(cor=0.70)_ = 0.60].

**Conclusions:**

The findings suggest that adolescent digital addiction is characterized centrally by restriction intolerance and withdrawal anxiety rather than mere usage gratification. Interventions targeting self-monitoring skills and building tolerance to disconnection may be strategically positioned to destabilize the psychopathological network.

## 1 Introduction

In the 21st century, the integration of digital technologies into the developmental ecology of adolescents has reached an unprecedented pace, establishing the state of being “permanently online” as a normative behavioral pattern. However, during adolescence a critical period for neurocognitive development this digitalization process entails serious psychopathological risks due to the “developmental mismatch” between the brain's reward-seeking systems (ventral striatum) and cognitive control mechanisms (prefrontal cortex) (Brand et al., 2016; Sengün et al., 2025). Adolescence is not only a period of neurobiological vulnerability but also a critical time for identity formation and peer connection (Crone and Konijn, 2018). Digital platforms tap directly into these developmental needs, turning social approval into measurable metrics. Because of this, adolescents often experience restricted access to their devices not simply as a loss of entertainment, but as a genuine threat to their social belonging. This combination of cognitive immaturity and psychosocial sensitivity makes adolescents a particularly important group for studying the structural dynamics of digital addiction. Characterized in the literature as “Real Captivity in a Virtual World” (Mutlu, 2025), this phenomenon manifests through various clinical presentations such as nomophobia (fear of being without a mobile phone), social media addiction, and smartphone addiction.

The most widely accepted theoretical framework for explaining the etiology of digital addiction is the Interaction of Person-Affect-Cognition-Execution (I-PACE) model. Developed by (Brand et al. 2016, 2019), this model posits that addictive behavior results from an individual's genetic/personality traits (P), affective (A) and cognitive (C) responses toward the internet, and a reduction in executive functions (E). Research focusing on the “execution” component of this model consistently indicates that self-regulation failure and a deficit in inhibitory control are strongly associated with digital addiction in adolescents (Tang and He, 2025). For instance, a study conducted by (Sengün et al. 2025) on Turkish adolescents found that technology addiction is directly associated with impairments in executive function skills, and that deficits in attentional control predict addictive behavior. In this framework, I-PACE components are treated as item communities (e.g., self-regulation and self-efficacy) whose conditional associations can be examined without assuming a single latent driver. This allows for a detailed examination of how specific cognitive processes (e.g., self-efficacy beliefs) functionally interact with specific executive deficits (e.g., regulatory failure) within the system.

Consistent with this functional perspective, within the I-PACE framework, while Self-Efficacy is traditionally categorized as a cognitive-affective construct (part of the “Person” or “Cognition” component), strictly focusing on its functional role reveals its critical link to the “Execution” system. Specifically, in the context of digital addiction, self-efficacy does not merely act as a static belief, but as a dynamic regulatory resource. The mechanism underlying this functional shift is that cognitive confidence acts as an essential precursor for executive control. According to Social Cognitive Theory, high self-efficacy is the prerequisite for initiating inhibitory control mechanisms under stress (Bandura, 1991). Without this cognitive-affective foundation, the executive system fails to mobilize against addiction impulses. Therefore, in this study, we conceptualize self-efficacy as a functional extension of the “Execution” component, as it provides the necessary cognitive framework that structurally co-occurs with self-regulation and resist impulsive urges.

However, the vast majority of studies in the current literature rely on traditional statistical methods (e.g., regression, structural equation modeling) that treat digital addiction and related psychological resources (self-efficacy, self-regulation) as “latent variables” and analyze them via sum-scores (Tang and He, 2025; Terzi et al., 2024). This approach presumes the diagnosis of “addiction” to be a homogeneous construct and masks the unique interactions between specific symptoms (e.g., “loss of control” vs. “withdrawal”). Conversely, Network Theory conceptualizes psychopathology not as the consequence of a latent cause, but as a dynamic system wherein symptoms mutually reinforce one another (Borsboom, 2017; Fried and Cramer, 2017). Grounded in the works of (Borsboom 2017) and (McNally et al. 2015), this perspective holds that a disorder is not the sum of its symptoms, but rather the network of interactions among them.

Recent studies have underscored the necessity of this methodological paradigm shift in the field of digital addiction. For example, (Al-Mamun et al. 2025) identified “daytime dysfunction” as a bridge symptom linking social media addiction to sleep quality, while (Zhang et al. 2025) mapped the comorbidity between internet addiction, depression, and insomnia. Furthermore, (Ren et al. 2025) highlighted that adolescents possess a more fragile network structure compared to adults, making this developmental period critical for investigation. However, a critical limitation of these pioneering network studies is their predominant focus on the “pathological interplay” between addiction and other deficits (e.g., distress, sleep disorders). This perspective often neglects the “protective architecture” of cognitive resources. Specifically, how adaptive mechanisms like self-regulation and self-efficacy are inversely related to specific addiction symptoms remains largely unmapped. Given that the I-PACE model explicitly positions reduced executive control as a core determinant of addiction (Brand et al., 2019; Sengün et al., 2025), excluding these cognitive resources leaves the “inhibitory” side of the psychopathological network incomplete.

To address this theoretical gap and provide a detailed structural analysis, the current study aims to map the specific associations between addiction symptoms (Nomophobia, Social Media Addiction) and cognitive resources (Self-Regulation, Self-Efficacy). While existing research has begun to map these structures, few studies have rigorously tested the structural stability and internal cross-validation of these networks within large adolescent cohorts (Epskamp et al., 2018; Fried and Cramer, 2017). Therefore, this study employs a split-half cross-validation strategy (Discovery vs. Validation) to ensure that the identified “central” symptoms and critical “bridge” connections are not statistical artifacts, but robust psychopathological targets for preventive interventions.

Mapping this “protective architecture” alongside pathological symptoms is a clinical necessity, not just a methodological exercise. This study aims to shift the focus from deficit-based addiction models to resilience-oriented network mapping. By integrating cognitive resources directly into the symptom-level network, we can identify specific, modifiable cognitive “nodes” rather than broad latent traits. Pinpointing exactly which self-regulatory skills or efficacy beliefs neutralize specific withdrawal symptoms provides empirical grounds for targeted interventions. Ultimately, this empowers clinicians to move beyond generic, often ineffective “digital detox” strategies (Radtke et al., 2022) and develop precision-targeted protocols to actively dismantle the psychopathological network from within (Borsboom, 2017).

Although regularized network estimation is primarily exploratory, we derived three specific hypotheses from the I-PACE framework and existing literature:

- **Hypothesis 1 (H1):** Digital addiction symptoms and cognitive resources will form distinct network communities connected primarily by negative bridges, reflecting an inverse association between cognitive control and addiction nodes.
- **Hypothesis 2 (H2):** Symptoms related to emotional distress—specifically withdrawal and restriction intolerance—will exhibit high centrality, functioning as core hubs within the network.
- **Hypothesis 3 (H3):** The overall network topology, including global connectivity and specific edge weights, will demonstrate structural invariance across the cross-validation subsamples.

## 2 Method

The study sample consisted of adolescents aged 14–18 years (*M* = 16.07, SD = 1.01) enrolled in vocational high schools in a province located in the Central Black Sea region of Türkiye. Data were collected from four vocational high schools, covering 37 classrooms across grades 9 through 12. A stratified random sampling approach was used to approximate the demographic composition of the participating schools. Stratification was based on grade level (9th−12th grades) and gender. Within each school, student lists obtained from classroom rosters were organized according to these strata, and participants were randomly selected using proportional allocation to reflect the actual grade and gender distribution of the schools. This procedure aimed to reduce selection bias and ensure that different stages of adolescence were adequately represented in the final analytical sample (*N* = 1,497). The data collection process was conducted between April and August 2024, and informed consent was obtained from participants and their legal guardians prior to administration. The study was found to be in accordance with the ethical standards of the Declaration of Helsinki via decision number 13.51 (dated August 22, 2025) of the Tokat Gaziosmanpaşa University Social and Human Sciences Research Ethics Committee.

To ensure data quality and mitigate selection bias, a multi-stage data screening process was conducted on the initial pool of respondents (*N* = 1,564). First, careless responding was assessed using the “longstring” index to detect invariant response patterns (e.g., selecting “3” for all items). Participants exceeding two standard deviations on this index were flagged as “straight-liners” and removed (*n* = 42). Second, multivariate outliers were identified using Mahalanobis distance with a conservative threshold of *p* < 0.001 (χ^2^ > 86.6 for 46 degrees of freedom). This procedure identified and excluded 25 cases that exhibited statistically improbable response combinations. Following these exclusions, the dataset was checked for missing values, and none were found. The final analytical sample consisted of 1,497 adolescents.

To enhance the internal consistency of the study and assess the internal consistency of the estimated network structure, a “split-half cross-validation” strategy was employed (Epskamp et al., 2018). Accordingly, the main dataset comprising 1,497 participants was randomly split into two equal subsamples:

- Discovery Group (*n* = 748): Network structure estimation and centrality analyses were performed on this group.
- Validation Group (*n* = 749): This group was used to test the stability and structural invariance of the obtained model.

The demographic characteristics (age, gender distribution, etc.) of both groups are presented comparatively in Table 1.

**Table 1:** Demographic characteristics of participants and comparison between groups.

Variables	Discovery group (*n* = 748)	Validation group (*n* = 749)	Test statistic	*p*
Age (Mean ± SD)	16.07 ± 1.01	16.06 ± 1.05	*t* = 0.25	0.80
Gender	–	–	*X*^2^ = 0.00	0.98
Female, *n* (%)	463 (61.9%)	465 (62.1%)		
Male, *n* (%)	285 (38.1%)	284 (37.9%)		

SD, standard deviation.

As shown in Table 1, independent samples *t*-test and chi-square analyses revealed no statistically significant differences between the Discovery and Validation groups in terms of age [*t*_(1, 495)_ = 0.25, *p* = 0.80] and gender distribution [$x_{\left(1\right)}^{2}$ = 0.00, *p* = 0.98]. These findings indicate that the randomization procedure successfully yielded demographically homogeneous subsamples.

## 3 Measures

### 3.1 Bergen social media addiction scale (BSMAS)

Developed by (Andreassen et al. 2012), this instrument consists of six items. The internal consistency coefficient of the original scale was calculated as 0.88. The BSMAS was adapted into Turkish by (Demirci 2019). Following adaptation, the unidimensional structure of the instrument was found to be acceptable (χ^2^ = 6.02, *df* = 9, *p* = 0.07; CFI = 0.95; TLI = 0.92; SRMR = 0.05; RMSEA = 0.08). In the current study, internal consistency was found to be 0.77 for Cronbach's Alpha and 0.78 for McDonald's Omega.

### 3.2 Self-efficacy scale for children

Developed by (Muris 2001) and adapted into Turkish by (Telef and Karaca 2012), this instrument consists of 21 items across three sub-dimensions: academic, social, and emotional. In the adaptation study, the three-factor structure was shown to possess acceptable goodness-of-fit values (RMSEA = 0.05, CFI = 0.96, GFI = 0.94, SRMR = 0.07). The internal consistency coefficient was calculated as 0.86 in the adaptation study. Rated on a five-point Likert scale (1 = Not at all, 5 = Very well), higher scores indicate higher levels of self-efficacy. In the current study, internal consistency was found to be 0.83 for Cronbach's Alpha and 0.83 for McDonald's Omega.

### 3.3 Self-regulation scale for adolescents

Developed by (Kaşikci and Ögülmüş 2023) to measure adolescents' general self-regulation skills, this instrument has a unidimensional structure and comprises 11 items. In the development study, it was determined that the single-factor structure explained 51% of the total variance, and Confirmatory Factor Analysis (CFA) results indicated good model fit (χ^2^/df = 4.55, CFI = 0.93, TLI = 0.91, SRMR = 0.04, RMSEA = 0.09). In the original study, the internal consistency coefficient (Cronbach's Alpha) was calculated as 0.90, and test-retest reliability as 0.78. The scale is rated on a five-point Likert type (1 = Strongly Disagree, 5 = Strongly Agree) and contains no reverse-coded items. Scores range from 11 to 55, with higher scores indicating higher levels of adolescent self-regulation. In the current study, internal consistency was found to be 0.81 for Cronbach's Alpha and 0.81 for McDonald's Omega.

### 3.4 Firat nomophobia scale

Developed by (Kanbay et al. 2022) to determine nomophobia (fear of being without a mobile phone) levels in the general population, this instrument has a unidimensional structure and consists of eight items. In the development study, the single-factor structure explained 55.9% of the total variance, and CFA results indicated perfect model fit (CFI = 0.99, TLI = 0.99, SRMR = 0.05). In the original study, the internal consistency coefficient (Cronbach's Alpha) was calculated as 0.89. Rated on a five-point Likert type (1 = Not appropriate at all, 5 = Completely appropriate), the scale contains no reverse-coded items. Scores range from 8 to 40, with higher scores indicating higher levels of nomophobia. In the current study, internal consistency was found to be 0.87 for Cronbach's Alpha and 0.87 for McDonald's Omega.

### 3.5 Data preparation and node selection

In this study, an item-level network analysis approach was adopted to minimize potential information loss associated with aggregating psychological constructs into total scores or sub-dimensions, and to enable examination of associations among observed variables at a finer level of resolution (Borsboom, 2017). Although the employed scales were originally developed and validated within latent variable frameworks, the present network analytic strategy remains agnostic regarding latent causal structures. Item-level relations are modeled as conditional statistical dependencies, allowing investigation of how observed variables co-occur within the estimated network structure without presupposing a single underlying common cause (Fried and Cramer, 2017). Unlike dimensionality reduction methods, which are often necessitated by sample size constraints, the relatively large sample of the current study (*N* = 1,497) permitted estimation of all 46 items across four instruments within a single regularized model, supporting parameter estimation with acceptable statistical precision (Christensen et al., 2018; Fried and Cramer, 2017).

The nodes included in the analysis consist of items from the Social Media Addiction (six items), Self-Efficacy (21 items), Nomophobia (eight items), and Self-Regulation (11 items) scales. Examining these variables at the item level aims to uncover the specific pathways between digital addiction symptoms and cognitive control mechanisms.

In network analyses, ensuring that nodes are empirically distinguishable is critical for preventing the formation of spurious clusters and maintaining the reliability of the network's topological structure (Mullarkey et al., 2019). Accordingly, a “Goldbricker” analysis (topological overlap test) was conducted on the main dataset of 1,497 participants using the network tools package (Jones, 2017). The examination, performed on the precise correlation matrix derived from the large sample, revealed no significant bad topological overlap (*p* < 0.05) between any pair of items; thus, it was decided to include all 46 items as unique nodes in the model.

In the final stage of the data preparation process, to test the structural stability of the estimated network structure, the dataset (*N* = 1497) was randomly split into two equal subsamples (*n*1 = 748 and *n*_2_ = 749). This approach is considered one of the most established methods recommended in the literature to demonstrate that the discovered network topology is non-spurious and to test the structural invariance of the model (van Borkulo et al., 2023).

### 3.6 Statistical analyses

Data analysis was conducted using the R statistical software environment (Version 4.3.2; R Core Team, Vienna, Austria). The Psychometric Network Analysis approach was adopted to reveal the complex interaction patterns between variables. The analysis process was structured in four main stages:

### 3.7 Node predictability

To assess the practical relevance of the network connections, we estimated node predictability using the *mgm* package (Haslbeck and Waldorp, 2020). This metric (nodal *R*^2^) quantifies the proportion of variance in each node that is explained by its connections with all other nodes in the network, thereby indicating the extent to which a symptom is determined by the network structure itself.

### 3.8 Network estimation and visualization

A Gaussian Graphical Model (GGM) was employed to map the associations between variables within the Exploratory Set (Set A). In this model, nodes represent the individual items, while edges depict the partial correlation coefficients estimated from polychoric correlations (to account for the ordinal nature of the Likert-type data) between two nodes, controlling for the influence of all other nodes in the network. To eliminate spurious associations and obtain an interpretable, sparse network structure, the Least Absolute Shrinkage and Selection Operator (LASSO) regularization was applied in conjunction with the Extended Bayesian Information Criterion (EBIC) model selection using the qgraph package. The hyperparameter (γ) was set to 0.50, consistent with the standard recommendation for exploratory research (Epskamp and Fried, 2018).

### 3.9 Centrality and bridge analyses

To identify the most influential symptoms within the network, the Expected Influence (EI) centrality metric was utilized, as it accounts for the summation of both positive and negative associations among nodes (Robinaugh et al., 2016). Additionally, to identify key nodes that facilitate the transition between digital addiction symptoms (Social Media Addiction and Nomophobia) and cognitive control mechanisms (Self-Efficacy and Self-Regulation), Bridge Strength was calculated using the networktools package (Jones, 2017).

### 3.10 Network stability and accuracy

The reliability of the obtained network model was tested using the bootnet package. To estimate the accuracy of edge weights, non-parametric bootstrapping with 1,000 iterations was applied, and 95% confidence intervals (CI) were examined. The stability of centrality indices was assessed using the case-dropping subset bootstrap method. The Correlation Stability Coefficient (CS-coefficient) was targeted to be above 0.50 as an indicator of robust stability (Epskamp et al., 2018).

### 3.11 Network comparison and validation test

To evaluate the within-sample stability of the estimated network model, a two-step internal validation procedure was implemented. First, the Network Comparison Test (NCT) was conducted using 1,000 permutations (van Borkulo et al., 2023). As a fundamental dimension of this procedure, we assessed three core components: (1) Network Structure Invariance (to test overall topology), (2) Global Strength Invariance (to test overall connectivity), and (3) the Edge Invariance Test, which is strictly essential for determining whether specific edge weights (symptom-level pathways) differ significantly between the Discovery and Validation networks. Second, to rule out the potential confounding effects of demographic variables, a sensitivity analysis was performed. Specifically, we regressed all items on age and gender, and computed the correlation between the adjacency matrix of the original network and that of the network estimated from the residuals. This procedure aimed to confirm that the observed symptom interactions were intrinsic to the psychopathology rather than artifacts of demographic variance.

## 4 Results

### 4.1 Discovery group network analysis

**Figure 1:**
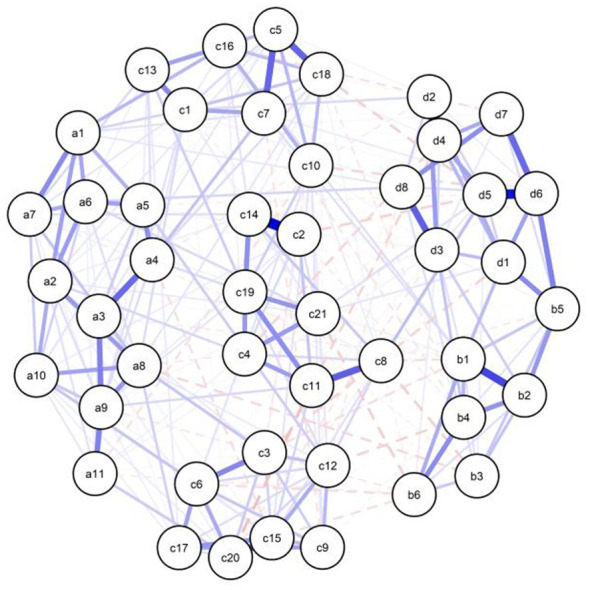
Network structure of the discovery sample (*n* = 748). Thickness of edges indicates partial correlation strength (max edge = 0.45). a1–a11: Self regulation scale item, b1–b6: Social media addiction scale item, c1–c21: Self efficacy scale item, d1–d8: Nomophobia scale item (based on polychoric correlations).

The Gaussian Graphical Model (GGM) estimated for the Discovery Group is visualized in Figure 1. To evaluate the topological clustering of the network objectively, we employed the Walktrap community detection algorithm. The analysis yielded a high Modularity index of 0.49, statistically confirming that the network is composed of distinct communities rather than a homogeneous structure. As indicated by this modularity partition, the “Cognitive Resources” domain separates into two statistical sub-clusters corresponding to Self-Regulation (nodes a1–a11) and Self-Efficacy (nodes c1–c21). While these clusters are topologically distinct, the adjacency matrix reveals consistent positive inter-community connectivity, exemplified by the edge connecting Self-Regulation item a1 to Self-Efficacy item c16 (*r* = 0.13), suggesting they operate as separate but complementary mechanisms.

Similarly, the “Digital Addiction” domain is organized into distinguishable communities for Nomophobia and Social Media Addiction, linked primarily by specific bridge symptoms; most notably, the connection between “withdrawal” (b5) and “restriction intolerance” (d6) exhibited the strongest intra-domain association (*r* = 0.22).

The interaction between these two opposing macro-communities (Resources vs. Addiction) is characterized almost exclusively by negative (dashed red) edges. For instance, a significant negative partial correlation was observed between Self-Efficacy (c21) and Social Media Addiction (b6) (*r* = −0.08). This structural pattern provides empirical evidence for an inverse association, suggesting that higher levels of cognitive resources statistically co-occur with reduced severity of digital addiction symptoms.

Furthermore, the visual topological placement of specific Self-Efficacy items is statistically corroborated by their centrality metrics. Specifically, node c11 (Z-Betweenness = 2.76) and node c19 (Z-Betweenness = 2.50) exhibited the highest intermediation capacity within the entire network. This structural metric confirms that these specific efficacy beliefs function as statistical “intermediary nodes”. Topologically, they occupy positions that may buffer the statistical associations between addiction symptoms and the broader psychological system.

Bridge Strength centrality identified the pivotal nodes facilitating the statistical transition between the “Digital Addiction” and “Cognitive Control” communities (see Table 2). Notably, the Social Media Addiction item b5 (“Feeling restless and troubled if forbidden from using social media”) displayed the highest bridge strength in the entire network (BS = 0.61). This finding identifies withdrawal symptoms (restlessness upon restriction) as the primary node associating addiction with cognitive resources. This suggests that the psychological distress caused by the *prevention* of access rather than the use itself is the critical mechanism that depletes self-regulation. Conversely, item a8 emerged as the most influential negative bridge node (BS = 0.47), indicating that self-monitoring acts as the central inverse correlate to addiction symptoms. Additionally, cognitive preoccupation (b1) and disconnection anxiety (d1) served as secondary bridges, reinforcing the anxiety centric nature of the network.

**Table 2:** Bridge centrality statistics of the most influential nodes.

Rank	Node	Item content (brief)	Community	Bridge strength
1	b5	Feeling restless if restricted from using SM (withdrawal)	SM addiction	0.61
2	b1	Preoccupation with social media	SM addiction	0.51
3	a8	Monitoring progress toward goals	Self-regulation	0.47
4	d1	Feeling restless when without phone	Nomophobia	0.47
5	a4	Expressing positive emotions	Self-regulation	0.43

### 4.2 Predictability

**Figure 2:**
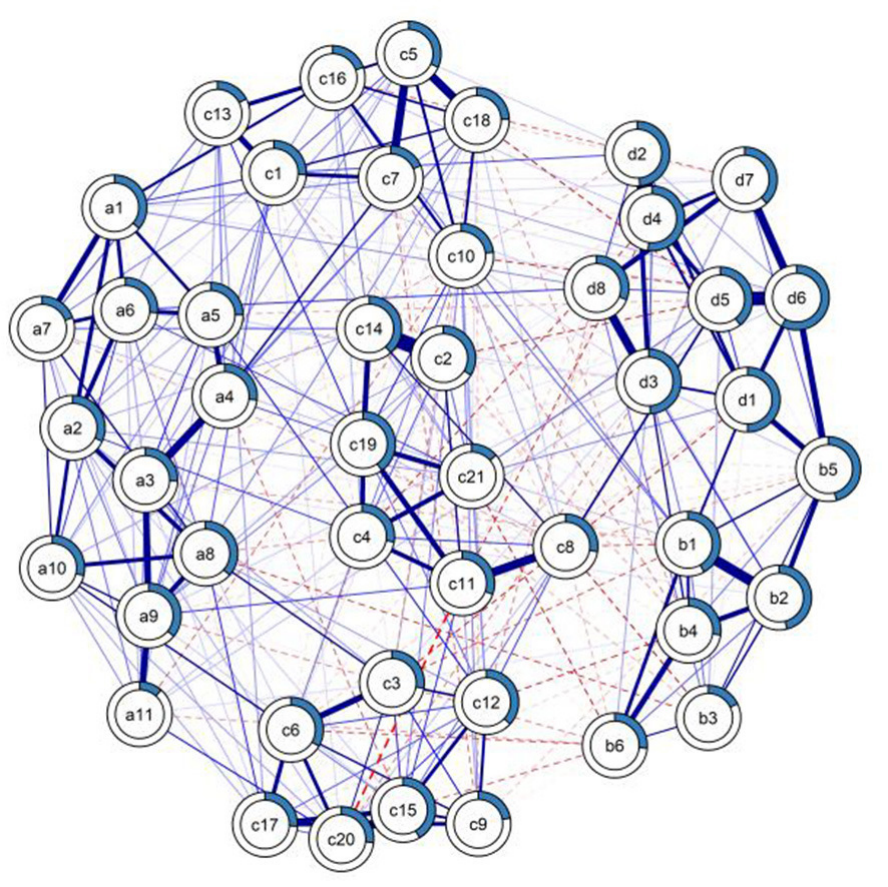
Predictability of nodes in the discovery group network.The blue ring around each node represents the proportion of explained variance (*R*^2^) for that specific symptom. Fuller rings indicate that the symptom is strongly predicted by other nodes in the network. a1–a11: Self regulation scale item, b1–b6: Social media addiction scale item, c1–c21: Self efficacy scale item, d1–d8: Nomophobia scale item.

To assess the extent of shared variance among symptoms, we examined nodal predictability (*R*^2^) (Figure 2). The network exhibited a mean predictability of 0.32, indicating that on average, 32% of the variance in each node is explained by its associations with neighboring nodes. While cross-sectional data precludes the confirmation of temporal directionality, this substantial level of explained variance suggests a highly interconnected symptom structure. It implies that the symptoms are not isolated manifestations but share a significant proportion of variance with their neighboring nodes within the network. Detailed analysis revealed that node d6 (“Feeling restless in places where mobile use is restricted”) had the highest shared variance (*R*^2^ = 0.58). This suggests that this specific manifestation of nomophobia is strongly associated with other network components. Similarly, the pivotal bridge node b5 (“Restlessness if restricted from using social media”) displayed substantial predictability (*R*^2^ = 0.46), indicating that withdrawal anxiety is deeply embedded in the network structure. In contrast, the protective bridge node a8 (“Monitoring progress toward goals”) maintained a higher degree of unique variance (*R*^2^ = 0.37). The fact that self-monitoring capability is less explained by the network structure suggests that it may be more independent and potentially more permeable to external psycho-educational interventions.

### 4.3 Centrality

**Figure 3:**
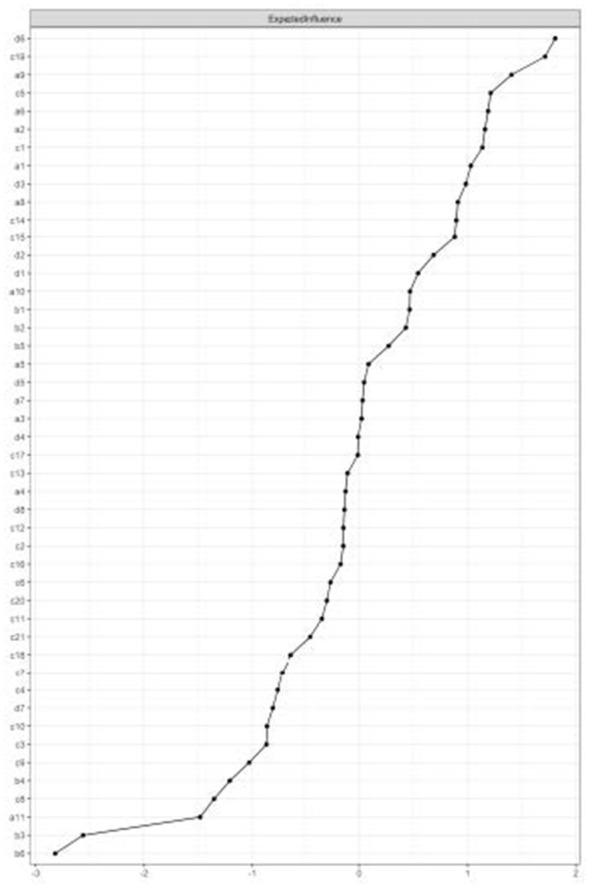
Centrality indices (expected influence) of the network in the discovery sample (*n* = 748). Nodes are ordered by their standardized *z*-scores. Higher values indicate greater influence on the overall network structure. d6: Nomophobia (Restriction intolerance), c19: Self-efficacy (thought suppression).

Importantly, centrality and bridge metrics do not possess universal cut-off values; their interpretation is inherently relative to the distribution of values within the estimated network. Accordingly, Expected Influence (EI) and Bridge Strength (BS) are interpreted based on nodes' comparative standing rather than absolute magnitude. To discern the network's architectural core, we examined Expected Influence (EI) centrality (Figure 3). The analysis identified the Nomophobia item d6 (“Feeling restless in places where mobile use is restricted”) as the network's most central component (EI = 1.81). The preeminence of this node suggests that the psychopathology is characterized less by the habitual act of scrolling through social media, and more by the acute anxiety associated with environmental restrictions. This implies that the primary stressor activating the network is not the usage itself, but the psychological intolerance of being “cut off” or restricted from access.

Counter-balancing this pathology, the protective systems exhibited structural resilience. The Self-Efficacy item c19 (“Ability to suppress bothering thoughts”) emerged as the second most influential node (EI = 1.72), followed closely by the Self-Regulation item a9 (“Keeping goal-relevant information in mind”) (EI = 1.40). The high centrality of these cognitive control nodes indicates that the network is not a passive system of addiction; rather, it reflects a robust mobilization of regulatory resources. The system appears to be actively engaging in thought suppression (c19) and working memory maintenance (a9) to attenuate the maladaptive impulses generated by restriction. In this hierarchy, Social Media Addiction symptoms functioned as downstream outcomes of this restriction-regulation conflict, rather than as primary instigators.

### 4.4 Cs-coefficient

**Figure 4:**
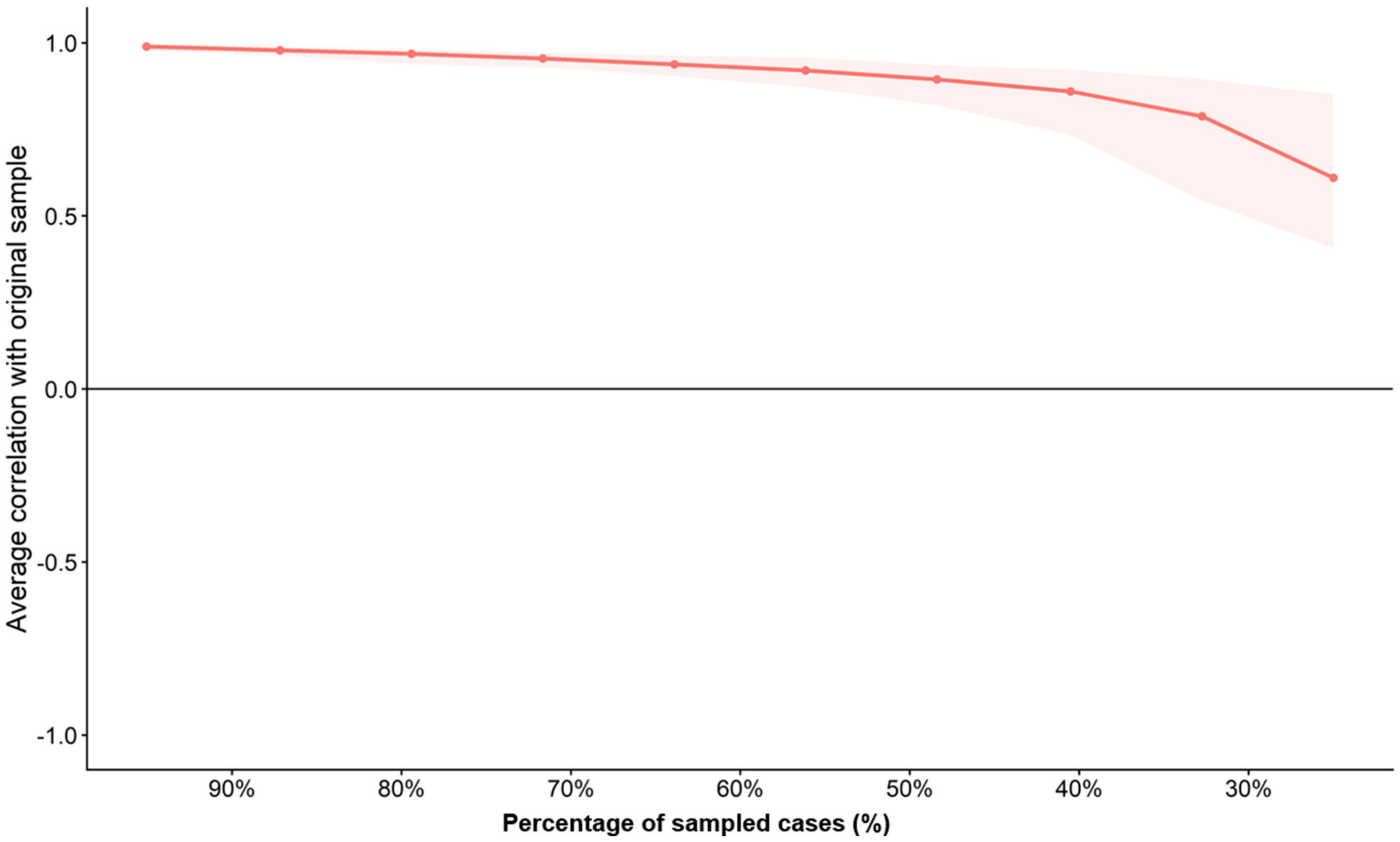
Network stability analysis based on case-dropping bootstrap (*n* = 748). The *x*-axis represents the percentage of cases dropped from the original sample, while the *y*-axis displays the average correlation between the centrality estimates of the original network and the subsamples. The red line depicts the stability of expected influence, which yielded a robust coefficient of CS_(cor = 0.70)_ = 0.60, indicating that the node ranking remains highly reliable even with significant data reduction.

To ensure that the identified centrality rankings particularly the dominance of restriction intolerance (d6) and thought suppression (c19) were not spurious artifacts of the specific sample composition, we conducted a rigorous case-dropping bootstrap analysis (1,000 iterations). The resulting stability profile is visualized in Figure 4.

The analysis yielded a Correlation Stability Coefficient CS_(cor = 0.70)_ of 0.60 for Expected Influence. This value substantially exceeds the stringent threshold of 0.50 recommended in the psychometric literature (Epskamp et al., 2018). In practical terms, this indicates that even if approximately 60% of the participants were randomly excluded from the dataset, the structural hierarchy of the most influential symptoms would remain statistically preserved with a correlation of at least 0.70. Consequently, the identification of intolerance of environmental restriction (d6) as the network's core component is a highly stable finding, resilient to sampling variations.

### 4.5 Edge weight stability (non-parametric bootstrap)

To evaluate the precision of the estimated network connections, we conducted a non-parametric bootstrap analysis with 1,000 resamples. This procedure generates 95% confidence intervals (CIs) for each edge weight, allowing us to assess whether the identified interactions are reliable or merely products of sampling error.

**Figure 5:**
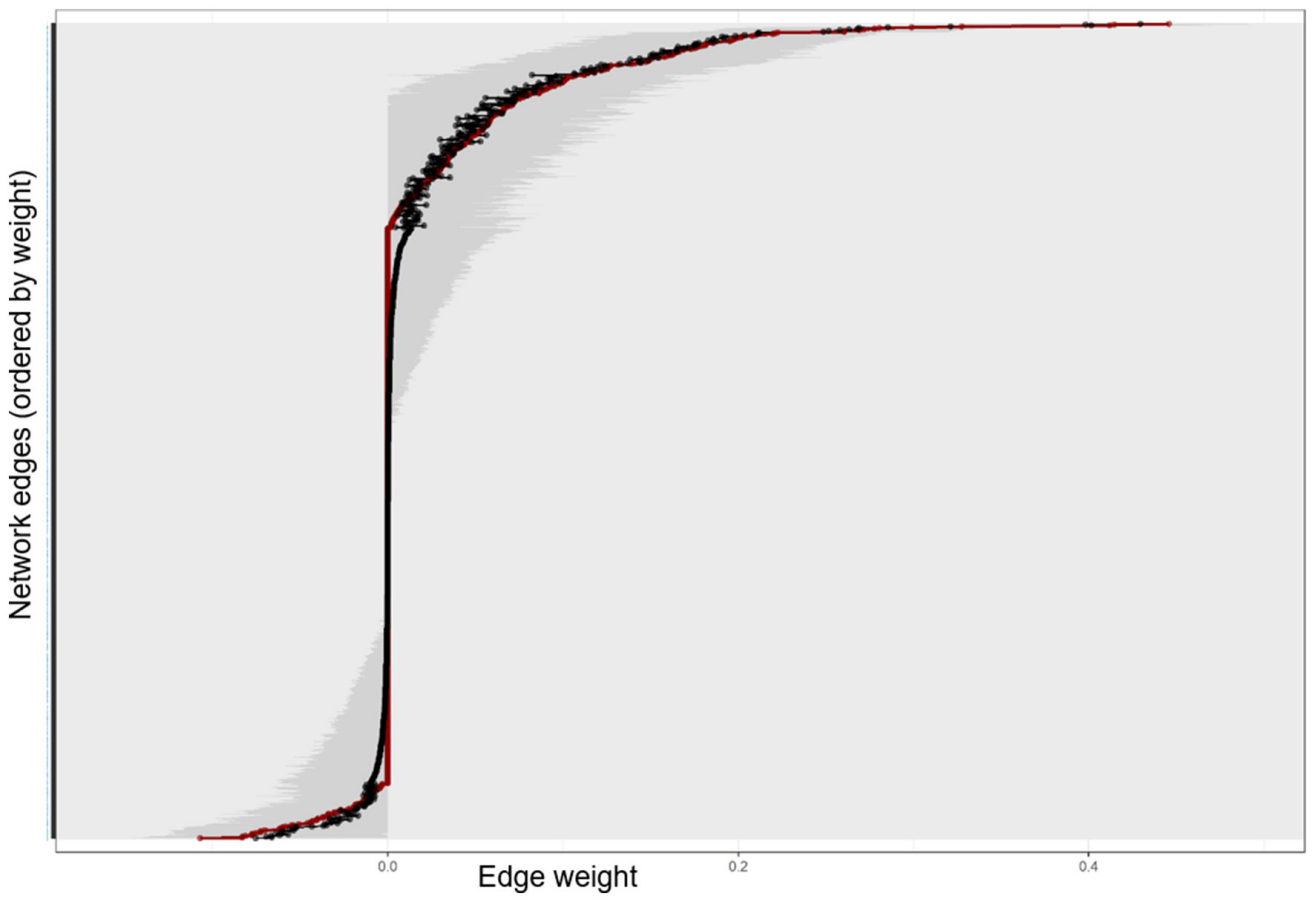
Edge weight stability analysis based on non-parametric bootstrap (*n* = 748). The red line represents the estimated edge weights from the original sample, while the black dots indicate the bootstrap means. The gray shaded area depicts the 95% confidence intervals (CIs). The *y*-axis lists all network edges ordered by weight. Narrow CIs indicate high precision and stability of the estimated connections.

As illustrated in Figure 5, the results demonstrate considerable stability across the network structure. The bootstrap means (black dots) align closely with the original sample estimates (red line), indicating minimal bias. The confidence intervals for the strongest edges such as the negative bridges between Nomophobia and Self-Regulation are notably narrow and do not overlap with zero. This confirms that the key pathways identified in the network, particularly the inhibitory links between cognitive resources and addiction symptoms, are statistically significant.

Finally, we conducted a sensitivity analysis to verify that the identified network structure was not attributable to demographic confounds. After regressing out the effects of age and gender from all items, the resulting network structure remained highly consistent with the original model (*r* = 0.98). This confirms that the observed symptom interactions are intrinsic to the psychopathology and are not artifacts of demographic variance.

### 4.6 Network comparison test (NCT)

**Figure 6:**
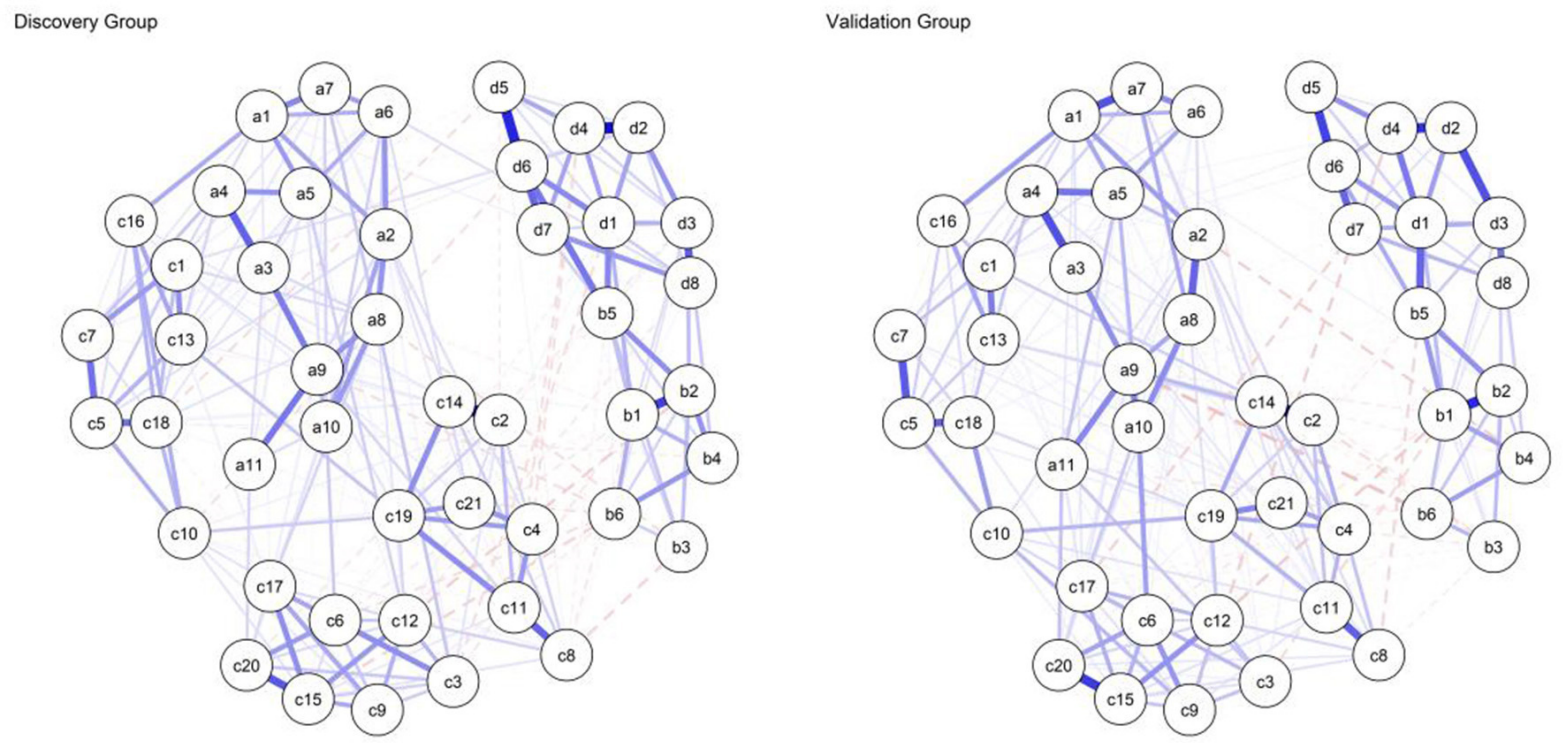
Visual comparison of the network structures in Discovery (*n* = 748, left) and Validation (*n* = 749, right) samples. The layouts are synchronized to facilitate direct visual comparison. The high degree of overlap in edge placement and thickness illustrates the structural invariance confirmed by the network comparison test (NCT, *p* > 0.05). a1–a11: Self regulation scale item, b1–b6: Social Media Addiction Scale Item, c1–c21: self efficacy scale Item, d1–d8: Nomophobia scale item.

To definitively establish the structural stability of the network model as an internal robustness check within the same cohort, we conducted a Network Comparison Test (NCT) with 1,000 permutations. This rigorous cross-validation procedure assessed whether the network topology identified in the Discovery Group (*n* = 748) remained invariant in the holdout validation subsample (*n* = 749) (see Figure 6). The NCT indicated no detectable differences in network structure between the two subsamples under the permutation framework. The test for Network Structure Invariance revealed no statistically significant difference in edge distributions between the two subsamples (*M* = 0.14, *p* = 0.20). As a central component of the NCT framework, we conducted an explicit Edge Invariance Test to examine whether individual edge weights differed significantly between the Discovery and Validation subsamples. The analysis demonstrated that, across all estimated parameters, no single edge weight differed significantly between the Discovery and Validation networks after applying the strict Holm-Bonferroni correction to control for multiple comparisons (all *p*-values >0.05). This robust non-significant result definitively confirms that the specific, item-level interactions between digital addiction and cognitive resources are highly stable and successfully cross-validated within the holdout validation subsample. Finally, the Global Strength Invariance test confirmed that the overall connectivity density of the network was preserved across groups (*S* = 0.47, *p* = 0.60).

## 5 Discussion

The present study employed a large-scale, split-half cross-validation strategy to map the specific symptom-level interactions between digital addiction and psychological resources among adolescents. By moving beyond the traditional latent variable approach, we examined the specific architecture of this psychopathological network. The findings not only confirmed the structural stability and invariance of the network (*p* > 0.05 in NCT; CS = 0.60) but also highlighted specific symptoms namely “discomfort from information deprivation” (d6) and “withdrawal upon prohibition” (b5) as the critical central elements of the system. These results offer empirical evidence consistent with the I-PACE model (Brand et al., 2019) and delineate the precise pathways through which cognitive resources counteracting addictive behaviors (Sengün et al., 2025).

### 5.1 The central feature: intolerance of restriction

A pivotal finding of this study is the identification of the nomophobia symptom d6 (“Feeling restless in places where mobile use is restricted”) as the most central node (EI = 1.81) and the most predictable element (*R*^2^ = 0.58) of the network. This result challenges the conventional view that social media addiction is primarily rooted in social interaction needs or the “Fear of Missing Out” (FoMO) (Przybylski et al., 2013). Instead, our findings suggest that for modern adolescents, the core psychopathology is rooted in “environmental restriction intolerance” the acute distress associated specifically with external prohibitions or lack of access. Within the framework of the I-PACE model, this symptom likely represents a maladaptive cognitive response (C-component) that is linked to the urge to use digital devices to regulate negative affect (Brand et al., 2016). The high predictability of this node further indicates that it is not an isolated symptom but a cumulative outcome of the activated addiction network, acting as a “central hub” that consolidates the entire maladaptive system (Borsboom, 2017). Node d6 exhibited the highest Expected Influence within the estimated network, indicating that it maintains the strongest level of conditional connectivity relative to other nodes. This pattern suggests that restriction-related discomfort occupies a structurally prominent position in the network topology. Such a configuration is consistent with recent network studies of digital addiction and nomophobia in adolescent populations (Ren et al., 2025), where restriction-related symptoms similarly emerged as highly central components.

### 5.2 Withdrawal: the bridge between deficit and addiction

Our bridge centrality analysis identified the Social Media Addiction item b5 (“Feeling restless and troubled if forbidden from using social media”) as the strongest bridge (BS = 0.61) connecting the addiction cluster to the cognitive resource cluster. Statistically, this indicates that the association between regulatory deficits and addictive behavior is primarily mediated by withdrawal symptoms rather than by the intensity of social media use itself. This prominence of withdrawal (b5) aligns with the “Compensatory Internet Use Theory” (Kardefelt-Winther, 2014) and the “Self-Medication Hypothesis” (Khantzian, 1997), suggesting that adolescents with lower self-regulation do not necessarily use social media for enjoyment, but rather because they cannot tolerate the negative affect arising from restriction. Thus, the gateway to addiction appears to be the failure to withstand disconnection (Tang and He, 2025).

### 5.3 The protective mechanism: self-monitoring and thought suppression

In contrast to the anxiety-centric addiction pathways, the network revealed a *protective* structure characterized by metacognitive monitoring. The Self-Regulation item a8 (“Monitoring progress toward goals”) and the Self-Efficacy item c19 (“Thought suppression”) emerged as key protective nodes. This suggests that “Self-Monitoring” the metacognitive ability to track one's own behavior and goals constitutes the primary line of defense against digital addiction (Hofmann et al., 2012). Adolescents who actively monitor their progress are likely better equipped to notice the early signs of “loss of control” and intervene before withdrawal symptoms (b5) activate the full addiction cycle. Furthermore, the ability to suppress bothering thoughts (c19) acts as a regulatory mechanism, attenuating the impact of the anxiety of restriction (d6) on the broader network (Sengün et al., 2025).

Contextualizing these structural findings within the daily lives of adolescents clarifies the real-world implications of the network's core symptoms. The central nodes of “restriction intolerance” (d6) and related “withdrawal” (b5) are practically associated with parent-adolescent conflict and school disciplinary issues. From a developmental perspective, peer validation and social belonging are critical needs; thus, high school students typically view digital devices not merely as entertainment, but as essential tools for social integration (Odgers and Jensen, 2020). Contextually, parental or school-imposed device restrictions are rarely perceived simply as rule enforcement; rather, they are experienced as threats of social isolation. This dynamic provides a conceptual background for the acute anxiety often linked to external restrictions. Conversely, the network's strongest protective bridge, “self-monitoring” (a8), reflects an adolescent's capacity to autonomously balance academic responsibilities with online social desires, independent of continuous adult oversight (Duckworth et al., 2019). Ultimately, these findings suggest that approaches to adolescent digital addiction could benefit from strengthening internal self-monitoring mechanisms, whereas relying solely on external prohibitions conceptually aligns with the network's core pathology.

### 5.4 Methodological reliability and cross-validation

A distinct strength of this study lies in its methodological rigor. As an internal validation check within the same cohort, we implemented a split-half cross-validation design to evaluate whether the estimated network structure and key node metrics were robust to random partitioning of the data. The Network Comparison Test (NCT) demonstrated that the network structure and global strength were invariant across Discovery and Validation groups (*p* > 0.05), confirming that the reported interactions are not idiosyncratic artifacts of a specific sample (van Borkulo et al., 2023). Furthermore, the sensitivity analysis (*r* = 0.98) ruled out demographic confounders, and the high stability coefficient (CS = 0.60) exceeded standard thresholds (Epskamp et al., 2018). These checks collectively establish the current network model as a reliable reference for understanding adolescent digital addiction.

#### 5.4.1 Limitations and future directions

Despite its large-scale and robust methodology, this study is not without limitations. First, although Network Theory conceptualizes psychopathology as a causal system, the cross-sectional nature of our data limits our ability to strictly determine the temporal directionality of edges; thus, the identified pathways should be interpreted as potential causal candidates to be verified in future longitudinal designs. Consequently, while centrality metrics suggest influence (Fried and Cramer, 2017), longitudinal data are required to definitively confirm whether withdrawal symptoms (b5) temporally precede the collapse of self-monitoring (a8). Future studies should employ Experience Sampling Methods (ESM) to capture these dynamics in real-time. Second, although we controlled for age and gender, unmeasured variables such as psychiatric comorbidity (e.g., ADHD) or family dynamics could influence the network structure. Finally, the study relied on self-report measures, which may be subject to social desirability bias, although the large sample size mitigates this risk to some extent. Additionally, although the stability coefficient (CS = 0.60) indicates a robust core structure, the estimation of a 46-node network with the current sample size may limit the statistical power to reliably detect very weak edges, despite the use of LASSO regularization.

**Clinical, Policy, and Research Implications:** the detailed architecture of the estimated network offers specific, “precision-oriented” interventions that diverge from traditional abstinence-based approaches. While standard therapies typically target broad constructs (e.g., general self-regulation), the current network topology allows clinicians to prioritize the specific symptom-components that exert the highest statistical influence on the system. By identifying the unique topological roles of specific symptoms, we propose three network-informed strategies:

**The “Detox Paradox” (Targeting Bridge b5)**: traditional interventions often prescribe strict “digital detoxes” or device confiscation. However, our bridge analysis reveals that “Withdrawal/Restlessness upon Restriction” (b5) is the single most critical pathway (BS = 0.61) strongly associating the pathology with the cognitive system. This implies that abrupt prohibitions may paradoxically activate the network's strongest bridge, further compromising self-regulation. Therefore, clinicians should avoid strict bans and instead employ “Graduated Exposure to Disconnection.” This protocol involves short, voluntary periods of non-use to build tolerance to the anxiety of restriction without provoking the acute distress associated with forced deprivation.

**Metacognitive “Tripwires” (Leveraging Node a8)**: unlike generic “willpower” training, the network identifies “Monitoring progress toward goals” (a8) as the specific protective bridge node. This suggests that interventions should focus on “Metacognitive Monitoring”—training adolescents to recognize the *internal cues* (e.g., the specific feeling of boredom or anxiety) that precede the urge to scroll. Implementing “screen-time budgeting” apps that require active user logging can operationalize this specific node, turning passive consumption into an actively monitored behavior.

**Targeting the Central Hub (Node d6)**: since “Intolerance of Environmental Restriction” (d6) is the network's stable core (EI = 1.81), Cognitive Behavioral Therapy (CBT) must specifically target the catastrophic beliefs associated with being offline (e.g., “If I am offline, I will be socially erased”). Targeting this specific cognition is statistically more efficient than addressing general screen-time habits, as deactivating the central hub is likely to destabilize the entire psychopathological network.

**Policy and Technology Design**: these findings also provide practical context for educational policies and technology design. Given the central position of restriction intolerance (d6), absolute smartphone bans in schools might relate to elevated withdrawal anxiety if implemented without concurrent cognitive skill development. Therefore, educational frameworks could benefit from shifting focus away from strict prohibition toward fostering active self-monitoring (a8) and digital literacy. In a similar vein, software developers might consider interface designs that encourage metacognitive reflection, rather than relying exclusively on lock-out timers that conceptually align with the restriction intolerance observed in this network.

**Future Research Directions**: for future research, the stability of the current model highlights the utility of symptom-level approaches alongside traditional total-score evaluations. Subsequent studies could employ longitudinal network models and Ecological Momentary Assessment (EMA) to observe the real-time temporal interactions between specific withdrawal symptoms and cognitive resources. Examining the temporal ordering of addiction nodes and self-regulation variables would further clarify the micro-level dynamics of the I-PACE framework.

In conclusion, this study provides a detailed analysis of the digital addiction landscape in adolescents. By identifying withdrawal intolerance as the critical bridge and restriction anxiety as the central feature, we identify potential targets for symptom-specific interventions that address the specific mechanisms underlying the pathology.

## Data Availability

The datasets presented in this study can be found in online repositories. The names of the repository/repositories and accession number(s) can be found below: https://doi.org/10.7910/DVN/BGXBRO.
